# Inhibiting TLR7 Expression in the Retinal Pigment Epithelium Suppresses Experimental Autoimmune Uveitis

**DOI:** 10.3389/fimmu.2021.736261

**Published:** 2022-01-05

**Authors:** Sheng-Min Lo, Yih-Shiou Hwang, Chao-Lin Liu, Chia-Ning Shen, Wei-Hsin Hong, Wei-Cheng Yang, Meng-Hua Lee, Chia-Rui Shen

**Affiliations:** ^1^Department and Graduate Institute of Medical Biotechnology and Laboratory Science, College of Medicine, Chang Gung University, Taoyuan City, Taiwan; ^2^Graduate Institute of Biomedical Sciences, College of Medicine, Chang Gung University, Taoyuan City, Taiwan; ^3^School of Medicine, College of Medicine, Chang Gung University, Taoyuan City, Taiwan; ^4^Department of Ophthalmology, Lin-Kou Chang Gung Memorial Hospital, Taoyuan City, Taiwan; ^5^Department of Chemical Engineering, Ming Chi University of Technology, New Taipei City, Taiwan; ^6^Biochemical Technology R&D Center, Ming Chi University of Technology, New Taipei City, Taiwan; ^7^Genomic Research Center, Academia Sinica, Taipei, Taiwan; ^8^Center for Molecular and Clinical Immunology, Chang Gung University, Taoyuan City, Taiwan

**Keywords:** autoimmune uveitis, barrier function, EAU, IL-17, RPE, TLR7

## Abstract

Experimental autoimmune uveitis (EAU), a model of human uveitis, is an organ-specific, T cell-mediated autoimmune disease. Autoreactive T cells can penetrate the blood-retinal barrier, which is a physical defense composed of tight junction-linked retinal pigment epithelial (RPE) cells. RPE cells serve as antigen-presenting cells (APCs) in the eye since they express MHC class I and II and Toll-like receptors (TLRs). Although previous studies have shown that supplementation with TLR agonists exacerbates uveitis, little is known about how TLR signaling in the RPE contributes to the development of uveitis. In this study, we isolated the RPE from EAU mice, which were induced by active immunization (aEAU) or adoptive transfer of antigen-specific T cells (tEAU). The expression of TLRs on RPE was determined, and both aEAU and tEAU mice exhibited induced *tlr7* expression. The TLR7 agonist R848 was shown to induce aggressive disease progression, along with significantly elevated levels of the uveopathogenic cytokine IL-17. Furthermore, not only IL-17 but also R848 appeared to enhance the inflammatory response and to impair the barrier function of the RPE, indicating that TLR7 signaling is involved in the pathogenesis of EAU by affecting the behaviors of the RPE and consequently allowing the infiltration of autoreactive T cells intraocularly. Finally, local application of shRNA against TLR7 delivered by recombinant AAV effectively inhibited disease severity and reduced IFN-γ and IL-17. Our findings highlight an immunomodulatory role of RPE TLR7 in EAU development and provide a potential therapeutic strategy for autoimmune uveitis.

## Introduction

The blood-retinal barrier (BRB) is a physical barrier composed of a layer of tight junction-linked retinal pigment epithelial (RPE) cells that provides an impermeable shield to prevent cells and molecules from entering the tissue of the retina (1, 2). The BRB separates the inner side of the eyes from the blood circulation and contributes to maintaining immune privilege in the ocular environment. Immune privilege involves tolerance to the introduction of antigens and does not to elicit an unexpected immune response (3). Impairment of the BRB might cause the influx of immune cells; thus, these cells might recognize rare self-antigens and trigger autoreactivity in the eye (4, 5). Uveitis is one of the most common autoimmune diseases associated with ocular blindness and is responsible for 5-25% of legal blindness in the population (6). Patients with autoimmune uveitis tend to have strong major histocompatibility complex (MHC) associations and exhibit ocular infiltration of autoreactive T cells in response to retinal antigens such as interphotoreceptor retinoid-binding protein (IRBP), retinal arrestin and recoverin (7). Evidence suggests that the activation of autoreactive T cells specific to IRBP is cross-reactive with the gut microbiota (8). Corticosteroids are still the major therapeutic agent used to treat uveitis (9), although immunosuppressants (such as cyclosporin A, azathioprine and rapamycin) (10) and biologics (such as anti-TNFα therapy) (11) are also used as adjunctive therapies.

Experimental autoimmune uveitis (EAU) is an animal model of human uveitis. The actively induced EAU (aEAU) model is the most common animal model, in which C57BL/6 mice are actively immunized with the uveitogenic antigen peptide IRBP_1-20_ emulsified with complete Freud’s adjuvant (CFA) by subcutaneous injection in combination with intraperitoneal injection of pertussis toxin (PTX) to break the BRB (12). Autoimmune uveitis can also be induced by the adoptive transfer of autoreactive CD4^+^ T cells (13). In an adoptively transferred EAU (tEAU) model (14), the use of T cell inhibitors, including cyclosporine A and FK506 (15, 16), verified the essential role of autoreactive T cells in the development of uveitis. Two major uveopathogenic T cell subgroups, Th1 and Th17, are involved and exert different impacts on disease progression (17). In the early stage, the Th1-derived cytokine IFN-γ was thought to be the major uveopathogenic cytokine in naïve recipient animal models (18). In contrast, the Th17 response was shown to participate in the late phase of EAU (19). IL-17-secreting T cells exhibit a strong antigen-specific response (20), and anti-IL-17 antibody treatment attenuates EAU development (21).

In addition to uveopathogenic cytokines, Toll-like receptors (TLRs) might also be involved in uveitis development. Subretinal delivery of TLR agonists induced the ocular infiltration of leukocytes and triggered uveitis in mice (22), and subcutaneous injection of several TLR agonists exacerbated aEAU disease severity (23). Moreover, mice deficient in myeloid differentiation primary response gene 88 (Myd88), which is a downstream signal of TLRs, were completely resistant to the induction of aEAU (24). In other autoimmune diseases, Myd88 overexpression in dendritic cells (DCs) could result in systemic lupus erythematosus (SLE)-like disease (25). Stimulation of TLR2 and TLR4 could enhance the inflammatory response in rheumatoid arthritis (RA) (26), and the expression of TLRs was found to be associated with the incidence of inflammatory bowel disease (IBD) and the pathogenesis of Behcet’s disease (27, 28). TLRs are mainly expressed by antigen-presenting cells (APCs), which bridge innate and adaptive immunity (29). In the ocular environment, RPE cells serve as APCs, express MHC class I and II (30), and induce inflammatory cytokines such as IL-6, IL-8 and MCP-1 (31) through TLR signaling. In the current study, we sought to test the hypothesis that specific TLR signaling in RPE cells is able to regulate uveopathogenic inflammation in uveitis. The induced expression of TLR7 on RPE cells from EAU mice was observed, and its role in disease development was investigated by using agonists or shRNA-mediated inhibition. Additionally, we elucidated the effects of the TLR7 agonist R848 and uveopathogenic cytokine IL-17 on the production of the inflammatory cytokine IL-6 and the impairment of barrier function in RPE cells.

## Materials And Methods

### Animals

Wild-type C57BL/6 (B6) mice (6-7 weeks old) were obtained from the National Laboratory Animal Center, Taiwan, and kept in the animal facilities at Chang Gung University. Animal care and experiments were approved by and performed in accordance with the Institutional Animal Care and Use Committee of Chang Gung University, Taiwan.

### Actively Induced Experimental Autoimmune Uveitis in Mice

According to a previous study (12), the aEAU animal model was established on the day 0 by subcutaneous immunization of an emulsion containing 100 μl of human IRBP_1-20_ peptide (2 μg/μl) in 100 μl of CFA (Sigma-Aldrich), distributed over 4 spots on the tail base to flank. A single dose (100 ng) of intraperitoneal injection of PTX was used as a costimulatory adjuvant. Age- and sex-matched B6 mice that did not undergo immunization were used as disease-free controls. aEAU mice were sacrificed on the 12th or 28th day after disease induction. The eyes and spleen were harvested, and TLR expression and immune responses were analyzed.

### Adoptively Transferred Experimental Autoimmune Uveitis

Nylon wool-purified splenic T cells were isolated from day-12 aEAU mice and cultured in the presence of IRBP_1-20_ (10 μg/mL) for 2 days (14). The tEAU animal model was then established by intraperitoneal injection of IRBP-specific T cells (5×10^6^/mouse) into naïve 6-week-old B6 mice.

### Primary RPE Cell Isolation

The method was performed according to previous studies with modifications (32–34). After enucleation of infant mice (10-14 days old) or adult EAU mice, the eyeballs were cut into halves along the circumferential line to create a ciliary body-free posterior eyecup. The retinal layer was removed from the eyecup, which was pretreated with trypsin, and RPE tissue was scraped (**Supplementary Figure 1**) and minced into a single-cell suspension in Dulbecco’s modified Eagle’s medium (DMEM) (Gibco, Thermo Fisher Scientific) containing 10% (v/v) fetal bovine serum (FBS) (Gibco).

### Cell Culture and Culture Reagents

Primary RPE cells were cultured in DMEM supplemented with 10% FBS, 100 U/mL penicillin (Gibco), and 100 µg/mL streptomycin (Gibco). Splenocytes and splenic T cells were cultured in minimum essential medium α (α-MEM) (Gibco) containing 10% FBS, 55 nM β-mercaptoethanol (Gibco), 100 U/mL penicillin, and 100 µg/mL streptomycin. ID8 cells were maintained in DMEM with 3% (v/v) FBS and 1× ITS (Sigma-Aldrich, Merck) containing 5 μg/mL insulin and transferrin and 5 ng/mL sodium selenite. The IRBP_1-20_ (GPTHLFQPSLVLDMAKVLLD) peptide was synthesized by the Peptide Synthesis Core Facility of Academia Sinica, Taiwan.

### Gene and Protein Expression Analysis in RPE Cells

Total RNA was extracted from cultured primary RPE cells or RPE cells that were freshly isolated from EAU mice by the TRIzol (Invitrogen, Thermo Fisher Scientific) method. Reverse transcription and real-time quantitative PCR were performed by an iScript cDNA Synthesis Kit and iQ SYBR Green Supermix with a CFX Connect Real-Time PCR Detection System (Bio-Rad Laboratories). A total of 10 targets were analyzed, and the primer sets used were published elsewhere (35–37). The production of the proinflammatory cytokine IL-6 by RPE cells was analyzed by a Ready-Set-Go! ELISA kit (eBioscience, Thermo Fisher Scientific). Specific signaling protein levels were identified by MILLIPLEX^®^ multiplex assays for Luminex (Millipore, Merck) according to the manufacturer’s instructions.

### Electric Cell-Substrate Impedance Sensing (ECIS) Analysis

Primary RPE cells were seeded at a density of 7×10^4^ cells/well in ECIS 8W10E culture dishes. When the cell confluence reached 100% (≃16 hours), recombinant mouse IL-17 (PeproTech) or R848 was added to the RPE cultures. The level of impedance at low frequency (400 Hz) was monitored by an ECIS system (Applied BioPhysics), and the effects on barrier function during the entire culture period were determined according to the manufacturer’s instructions.

### Generation of Recombinant Adeno-Associated Viral (AAV) Vectors and rAAV-TLR7-shRNA

TLR7-specific shRNA (5’-GCCCTTTACCTGGATGGAAAC-3’) and scramble shRNA were subcloned into the pAAV-IRES-GFP plasmid (Stratagene) AAV vector and were named pAAV-shTLR7 and pAAV-scramble, respectively (**Supplementary Figure 2**). Recombinant AAV-8 virus production was performed with the AAV helper system by the Institute of Biomedical Sciences of Academia Sinica, Taiwan. Titers of rAAV-TLR7-shRNA (rAAV.shTLR7) and rAAV-scramble-shRNA (rAAV.sc) were determined by RT-qPCR analysis for green florescence protein (GFP) by calculating the viral genome copy number.

### Subretinal Treatment With R848 and rAAV-TLR7-shRNA

The TLR7 agonist R848 (4 μg/mouse) (Resiquimod; InvivoGen) and rAAV-TLR7-shRNA (4×10^10^ vg/mouse) were subretinally injected into both eyes of tEAU mice on day 0. tEAU mice treated with balanced salt solution (BSS) or rAAV-scramble shRNA served as controls. After 12 days of treatment, clinical disease severity was examined by fundoscopy as described previously (38, 39). Treated tEAU mice were sacrificed on the 28th day after disease induction. The eyes and spleen were harvested, and gene expression, histopathology and the immune response were analyzed *in vitro*.

### Disease Severity Scoring by Fundoscopy

From the 5th day to 28th day after disease induction, disease severity scoring was performed by fundoscopy every 3 weekdays; the final evaluation was conducted on the 28th day. After anesthetizing and dilating the pupil with proparacaine 0.5% and tropicamide 1% (ALCON), the clinical score of uveitis in each eye was graded based on the degree of cell infiltration as published elsewhere (38, 39). The severity score was graded from 0 to 4 as follows: 0, no change; 0.5 to 3, few focal lesions to a pattern of linear lesions, increasing vasculitis, neovascularization, retinal hemorrhage, and papilledema; and 4, large retinal detachment and retinal atrophy.

### Histopathology

The eyeballs were collected and fixed in 3.7% formaldehyde on the 28th day after tEAU induction. The fixed tissues were embedded in paraffin, sectioned at a thickness of 5 μm, and stained with hematoxylin and eosin (H&E) (Sigma-Aldrich).

### Immune Cell Phenotyping

Splenocytes were obtained from untreated tEAU mice, and samples containing 10^5^ cells were labeled with anti-CD4-PerCP (BD Biosciences) and anti-CD25-APC (eBioscience) to examine surface marker expression. Intracellular Foxp3 analysis was performed using anti-Foxp3-PE (eBioscience) and the Foxp3/transcription factor staining buffer set (eBioscience) according to the manufacturer’s instructions. Flow cytometric analysis was performed by an Attune NxT flow cytometer (Thermo Fisher Scientific), and the data were analyzed by FlowJo software (Clever.ly, BD Biosciences).

### IRBP-Specific Immune Responses

Splenocytes were isolated from the treated and untreated tEAU mice and cultured in the presence of IRBP_1-20_ (10 μg/mL) at a density of 4×10^6^ cells/well in 24-well plates for 4 days. The levels of the cytokines IFN-γ, IL-17, IL-10 and IL-6 were analyzed using Ready-Set-Go! ELISA kits (eBioscience, Thermo Fisher Scientific).

### Statistical Analysis

Nonparametric analyses, including the Mann-Whitney U test or Wilcoxon signed rank test, were performed by statistical tools in GraphPad Prism software. A statistically significant difference was defined as one with a p-value less than 0.05.

## Results

### TLR7 Expression Was Significantly Upregulated in Uveitis

**Figure 1:**
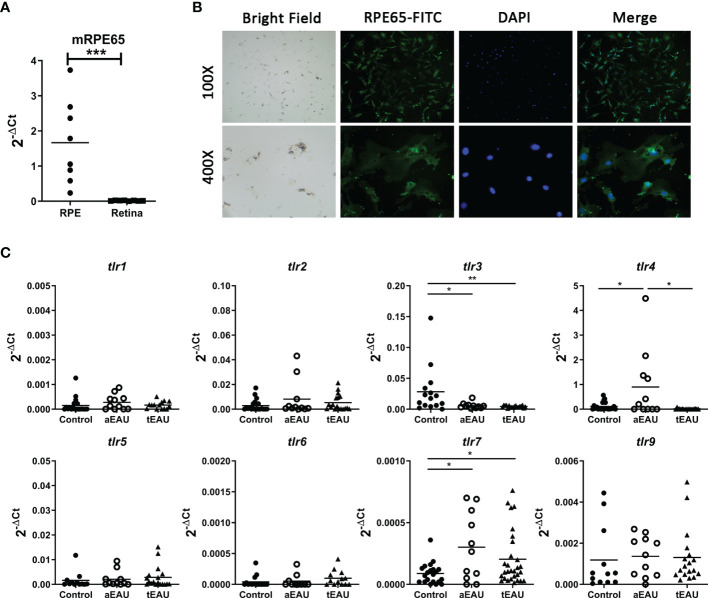
Significantly increased levels of tlr7 expression were found in freshly isolated RPE cells from aEAU and tEAU mice. **(A)** Primary RPE cells were isolated from adult C57BL/6 mice, RNA was extracted, and the expression of the RPE-specific molecule RPE65 was analyzed by RT-qPCR. Retinal layers, which are adjacent tissues in the retinal pigment epithelium, served as negative controls. **(B)** Primary RPE cells were isolated from young C57BL/6 mice (10-14 days old) and cultured to amplify their numbers until further use. The protein level of RPE65 on the cell surface was assessed by immunofluorescence with FITC-conjugated anti-RPE65, and the nuclei of cells were stained with DAPI. The results are displayed at magnifications of 100× and 400×. **(C)** C57BL/6 mice were induced to develop aEAU and tEAU by active immunization or adoptive transfer of IRBP-specific T cells, respectively. On the day of sacrifice, primary RPE cells were obtained from aEAU mice and tEAU mice, and RPE cells from healthy mice served as controls. RNA was purified and analyzed by RT-qPCR to quantify the expression levels of different TLRs (*p < 0.05, **p < 0.01, and ***p < 0.001).

TLRs seem to be expressed on RPE cells, so we hypothesized that TLR signaling is associated with the development of uveitis. First, primary RPE cells were freshly isolated from EAU mice, and TLR expression was analyzed. To ensure the purity of RPE cells, the expression of RPE65 was examined in freshly obtained RPE cells. The real-time RT-PCR results show that *rpe65* expression was significantly increased in freshly isolated RPE cells compared with the retina, which is the tissue adjacent to the retinal pigment epithelium (**Figure 1A**). The presence of RPE65 in these RPE cells was also confirmed by immunofluorescence analysis (**Figure 1B**).

Next, we examined which TLRs were involved in the development of EAU, and the expression levels of different TLRs were analyzed in RPE cells from EAU mice. Primary RPE cells were obtained from aEAU and tEAU animals and analyzed. **Figure 1C** shows significantly increased levels of *tlr7* expression in both aEAU and tEAU mice. However, RPE cells from only aEAU animals exhibited upregulated *tlr4* expression levels, indicating that the adjuvant in the aEAU induction protocol induced *tlr4* expression. This result suggests that the induced expression of *tlr7* but not *tlr4* contributes directly to the induction or development of EAU.

### The TLR7 Agonist R848 Exacerbated Disease Severity in EAU Mice

**Figure 2:**
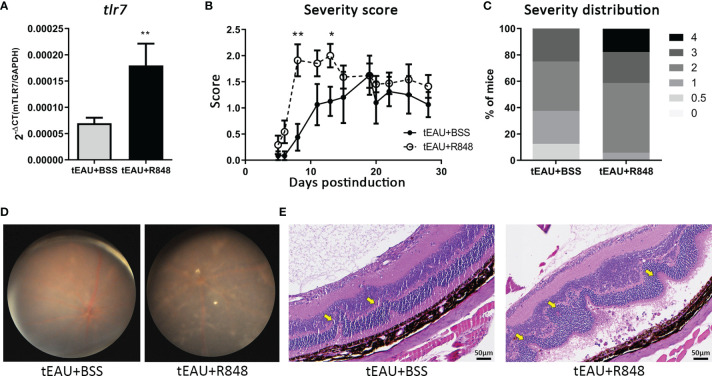
Subretinal delivery of the TLR7 agonist R848 induced TLR7 expression and exacerbated disease severity in the tEAU model. C57BL/6 mice were adoptively transferred with IRBP-specific T cells obtained from aEAU mice to induce tEAU. On the same day, tEAU mice were subretinally injected with the TLR7 agonist R848 (2 μg/eye) (tEAU+R848, n=17). The mice that received injections of BSS served as controls (tEAU+BSS, n=8). The eyes of these mice were monitored by fundoscopy and scored regularly. On the day of sacrifice, splenocytes and one eye from each mouse were harvested for autoimmunity and histological analysis. **(A)** Primary RPE cells were obtained from the other eye from ach tEAU mouse, and tlr7 gene expression in these RPE cells was determined by RT-qPCR. **(B)** Disease progression was monitored as scheduled, and the recorded disease score is shown as the mean ± SD. (*p < 0.05; and **p < 0.01). **(C)** The severity distribution of disease on day 28 before sacrifice was summarized among tEAU mice treated with or without R848. **(D)** Representative images showing fundoscopic analysis of tEAU mice treated with or without R848 on day 28 before sacrifice. **(E)** The eyes from the two groups of tEAU mice were fixed in 3.7% formaldehyde, sectioned and stained with H&E for ocular histopathological analysis (magnification: 200×).

To clarify whether TLR7 contributes to the development of autoimmune uveitis, the TLR7 agonist R848 was used to identify the effects of TLR7 on a mouse model of tEAU. On the day of tEAU induction, 6-week-old B6 mice received an intraperitoneal injection of IRBP-specific T cells (5×10^6^/mouse) and subretinal injection of R848 (4 µg/mouse). Healthy C57BL/6 mice without EAU induction were administered a subretinal injection of R848 and served as disease-free controls (data not shown). As shown in **Figure 2**, tEAU mice treated subretinally with R848, exhibited significantly higher *tlr7* gene expression in RPE cells than tEAU mice that received BSS treatment (**Figure 2A**). **Figure 2B** shows the disease severity curves of the mice receiving R848 or BSS as controls, and the severity score was determined by fundoscopy during the period of disease induction and development (38, 39). In tEAU mice that received R848 treatment, disease peaked at days 8-13 after induction, and severe disease lasted until the day of sacrifice. In contrast, in the absence of R848 treatment, disease peaked on day 19 in mice that received BSS treatment. The peak score in R848-treated mice was approximately 30% higher than that in BSS-treated control mice. **Figure 2C** summarizes the disease severity distribution in tEAU animals. Overall, 37.5% of BSS-treated tEAU mice developed mild disease (score ≤ 1), while the percentage was 5.8% in the R848-treated group. Moreover, 17.6% of R848-treated mice exhibited very severe disease (score=4). Fundoscopy (**Figure 2D**) and histological analysis (**Figure 2E**) showed many more infiltrated cells in the ocular fundus, along with neovascularization, disordered structures and significant morphological changes, in the R848-treated group, indicating that R848 exacerbated ocular inflammatory cell infiltration and impaired retinal tissue structure.

**Figure 3:**
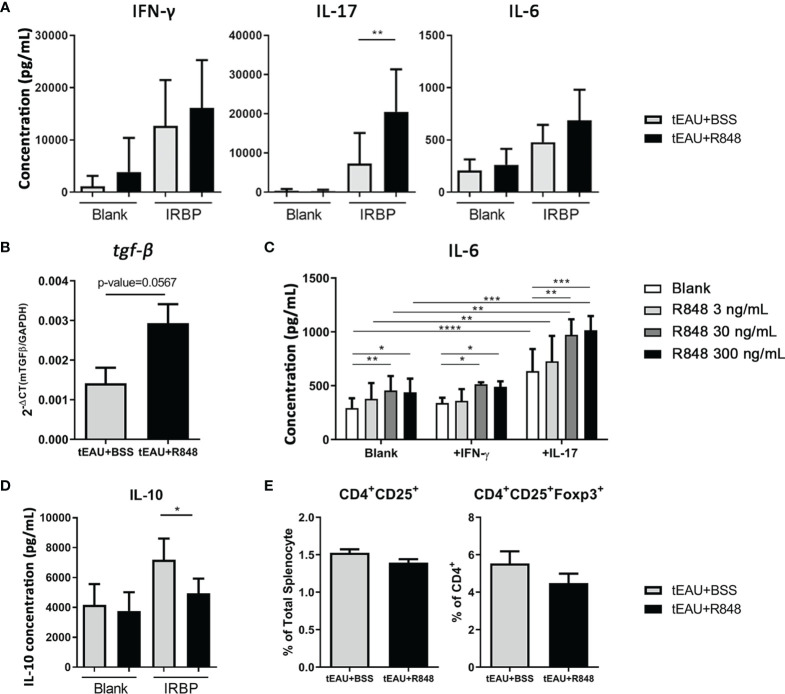
R848 enhanced the production of pathogenic and inflammatory cytokines. Splenocytes and RPE cells were freshly obtained from R848-treated tEAU mice (tEAU+R848, n=17) and control animals (tEAU+BSS, n=8) on the day of sacrifice. **(A)** Splenocytes were cultured with 10 μg/mL IRBP for 96 hours, and the levels of IFN-γ, IL-17 and IL-6 in the supernatant were measured by ELISA. **(B)** RNA was purified from primary RPE cells, and tgf-β gene expression was quantified by RT-qPCR. **(C)** Primary RPE cells were cultured and stimulated with a TLR7 agonist (R848) in the presence or absence of the uveitis-associated cytokines IFN-γ and IL-17. The culture supernatants were collected at 24 hours, and the levels of the proinflammatory cytokine IL-6 were measured by ELISA. **(D)** Splenocytes were cultured in to the presence of 10 μg/mL IRBP for 96 hours, and the levels of IL-10 in the culture supernatants were determined by ELISA. **(E)** The presence of Tregs was assessed by flow cytometric analysis of CD4^+^CD25^+^ or CD4^+^CD25^+^FoxP3^+^ T cells. These data are representative of at least three independent experiments and presented as the mean ± SD (*p < 0.05; **p < 0.01; ***p < 0.001; and ****p < 0.0001).

Treatment with R848 exacerbated ocular inflammation. To examine whether R848 affected antigen-specific immunity, the production of pathogenic cytokines associated with uveitis, IFN-γ and IL-17, and IL-6 by splenocytes in response to IRBP in R848-treated tEAU mice were compared with those from BSS-treated tEAU mice. The levels of the cytokines IFN-γ, IL-17 and IL-6 were relatively increased in R848-treated tEAU mice (**Figure 3A**). The IRBP-specific IL-17 level was significantly elevated. In addition, the gene expression of *tgf-β* was upregulated in RPE cells from the tEAU+R848 group compared to the BSS-treated group (**Figure 3B**). To examine the direct effects of R848 on RPE cells, we treated primary RPE cells with different doses of R848 and analyzed the production of IL-6, which is a proinflammatory cytokine that is produced in response to specific pathogen-associated molecular patterns (PAMPs). R848 (30 and 300 ng/mL) could significantly induce RPE cell production of IL-6. Moreover, IL-6 production was significantly increased when RPE cells were stimulated with both R848 and recombinant IL-17, revealing a synergistic effect (**Figure 3C**). In contrast, treatment with the TLR7 antagonist hydroxychloroquine sulfate (HCQ) (4000 ng/mL) significantly reduced IL-6 production. Although the addition of IL-17 enhanced IL-6 levels, it failed to restore the suppressive effect of HCQ on IL-6 production (**Supplementary Figure 3**).

### R848 Impaired Barrier Function in Primary RPE Cells

**Figure 4:**
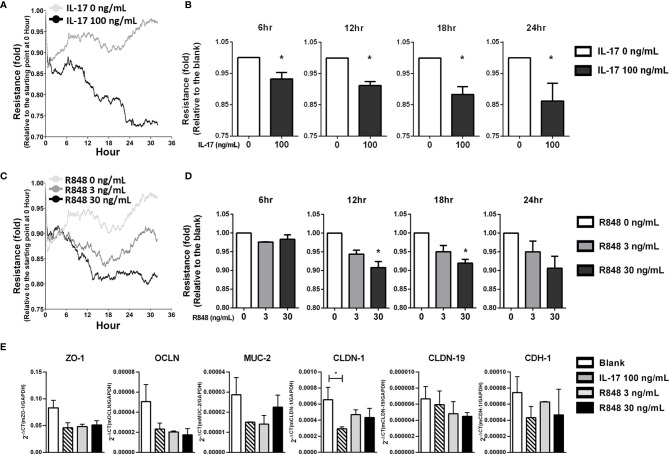
R848 plus IL-17 significantly impaired the barrier function of primary RPE cells *in vitro*. Primary RPE cells were isolated from young C57BL/6 mice (10-14 days old), expanded and cultured in ECIS 8W10E culture plates for functional analyses. **(A)** To determine the effects of IL-17 on barrier function in RPE cells, 0 or 100 ng/mL IL-17 was added to cultures containing 100% confluent RPE cells, and barrier function was examined by ECIS. The line graph shows the representative results. The X-axis indicates the time after stimulation with IL-17. The Y-axis indicates the fold change in the resistance level at each time point relative to 0 hours. **(C)** The ECIS assays were performed to identify the effects of R848, 0, 3 or 30 ng/mL R848 was added to the cultures, and ECIS analysis was performed according to the schedule. **(B, D)** The data in the bar chart were normalized to each corresponding blank (untreated) group. The data shown are representative of at least three experiments and presented as the mean ± SD (*p < 0.05). **(E)** Primary RPE cells were cultured and stimulated with IL-17 (100 ng/mL) or R848 (3 or 30 ng/mL) for 6 hours, RNA was isolated and the tight junction-associated gene expressions were examined by RT-qPCR. These data are representative of at least two independent experiments and presented as the mean ± SD. (*p < 0.05).

Because IL-17 can impair the expression of tight junction proteins in ARPE-19 cells (40), we next determined whether IL-17 combined with TLR7 signaling to affect the barrier function of RPE cells. Impedance was measured to quantify the integrity of the epithelial barrier by ECIS analysis. **Figure 4A** shows an instant decrease in impedance in primary RPE cells after treatment with recombinant IL-17, indicating an impairment of barrier function. Statistical analysis showed a significant difference between the IL-17-treated and untreated groups, with an approximately 10% decrease from 6 to 24 hours after treatment (**Figure 4B**). Likewise, R848 treatment also resulted in dysfunction of the epithelial barrier in primary RPE cells, and **Figure 4C** shows that R848 induced a dose-dependent reduction from 3 ng/mL to 30 ng/mL. Treatment with 30 ng/mL R848 reduced the impedance to 90% (**Figure 4D**). Moreover, under the stimulation of IL-17 or R848, the expression of tight junction-associated genes was down-regulated. In particular, treatment of IL-17 significantly reduced the expression of *claudin1* (**Figure 4E**). These results indicated that the TLR7 agonist R848 exacerbated disease by increasing inflammatory cytokine production and impairing barrier function. This result suggested that the increase in *tlr7* gene expression was associated with the exacerbation of uveitis.

### Subretinal Delivery of rAAV.shTLR7 Ameliorated Uveitis Severity

**Figure 5:**
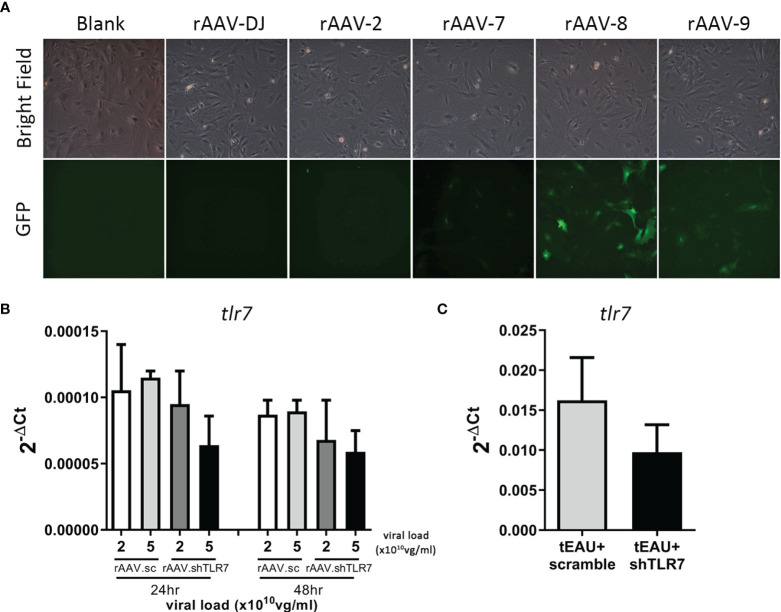
rAAV.shTLR7 inhibited tlr7 gene expression in primary RPE cells *in vivo* and *in vitro*. **(A)** Primary RPE cells were infected with different serotypes of rAAV-GFP (1×10^11^ vg/mL) (rAAV-DJ, rAAV-2, rAAV-7, rAAV-8, or rAAV-9), and GFP expression levels were determined to assess the infection efficiency of rAAVs by fluorescence microscopy. **(B)** Primary RPE cells were infected with 2 or 5 ×10^10^ vg/mL rAAV.sc or rAAV.shTLR7 for 24-48 hours, RNA was isolated, and tlr7 gene expression was determined by RT-qPCR. The data from two representative experiments are shown and presented as the mean ± SD. **(C)** C57BL/6 mice were adoptively transferred with IRBP-specific T cells, which were obtained from aEAU, mice to induce tEAU disease. On the same day, tEAU mice were subretinally injected with rAAV.sc or rAAV.shTLR7. On the day of sacrifice, the primary RPE layer was freshly obtained from the animals, and tlr7 gene expression was examined by RT-qPCR.

RNA interference is widely used to manipulate gene expression and is considered a powerful therapeutic tool. To investigate whether *tlr7* gene expression is directly associates with the induction of uveitis and serves as a potential therapeutic target, a recombinant AAV (rAAV) delivering an shRNA against TLR7 was used. Before performing the treatment, we first identified the appropriate serotype of rAAV with robust efficiency in primary RPE cells. rAAV-8 had the highest infection rate according to GFP expression, followed by rAAV-9 (**Figure 5A**). Next, rAAV.shTLR7 was constructed, and its inhibitory effect on TLR7 expression was examined. **Figure 5B** shows that infection with rAAV.shTLR7 dose- and time-dependently inhibited *tlr7* expression in primary RPE cells at a viral load of 2 or 5×10^10^ vg/mL at 24 or 48 hours *in vitro*. To assess the ability of rAAV.shTLR7 to downregulate TLR7 levels, RPE cells were isolated from tEAU mice that received rAAV.shTLR7, and TLR7 expression was compared with that in rAAV.sc-treated tEAU animals. The mice treated with rAAV.shTLR7 showed lower *tlr7* gene expression in the RPE layers than rAAV.sc-treated mice (**Figure 5C**).

**Figure 6:**
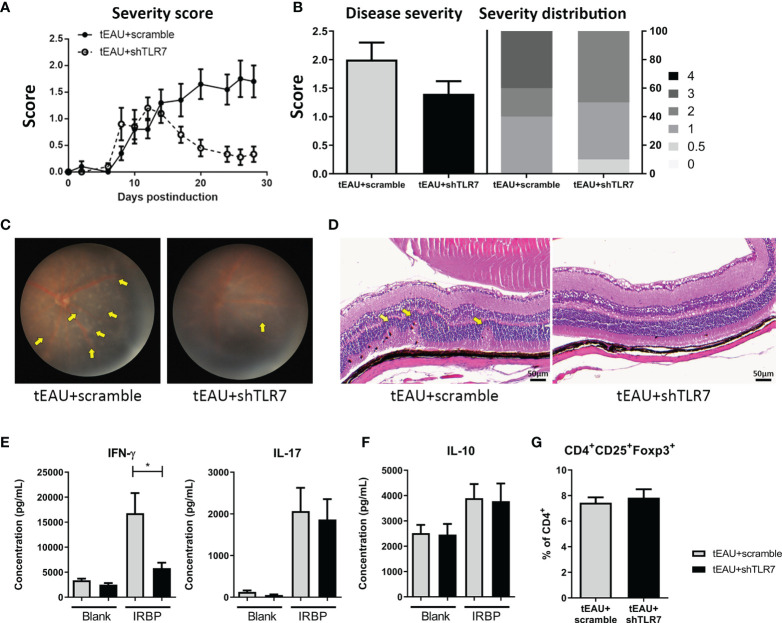
Subretinal delivery of rAAV.shTLR7 reduced disease severity in the tEAU model. C57BL/6 mice were adoptively transferred with IRBP-specific T cells obtained from aEAU mice to induce tEAU. On the same day, tEAU mice were subretinally injected with rAAV.shTLR7 (tEAU+shTLR7, n=10). The mice that received injections of rAAV.sc served as controls (tEAU+scramble, n=10). The eyes of these mice were monitored and scored regularly by fundoscopy. On the day of sacrifice, splenocytes and one eye from each mouse were harvested for autoimmunity and histological analyses. **(A)** Eyes of EAU mice were monitored by fundoscopy and scored regularly. Disease severity was monitored and the recorded disease score is shown as the mean ± SEM. **(B)** The left panel shows the disease severity, which indicates the most severe scores of individual mice during disease progression, and the right panel shows the disease distribution on day 28 among the tEAU mice treated with rAAV.shTLR7 or rAAV.sc. **(C)** The representative images showing fundoscopic analysis of tEAU mice treated with rAAV.sc (left) or rAAV.shTLR7 (right) on day 28 before sacrifice. **(D)** The eyes of tEAU mice in the two groups were fixed in 3.7% formaldehyde, sectioned and stained with H&E for ocular histopathological analysis (magnification: 200×). **(E)** Splenocytes were cultured in to the presence of 10 μg/mL IRBP for 96 hours, and the levels of the pathogenic cytokines IFN-γ and IL-17 and IL-6 in the supernatant were measured by ELISA. **(F)** The levels of IL-10 in the splenocyte culture supernatant with or without IRBP (10 μg/mL) were measured by ELISA. **(G)** Splenocytes were freshly isolated, and the phenotypes of Tregs (CD4^+^CD25^+^Foxp3^+^) were analyzed by flow cytometry. The data are presented as the mean ± SD. (*p < 0.05).

To evaluate whether rAAV.shTLR7 could treat autoimmune uveitis, on the day of tEAU induction, the mice were treated with rAAV (4×10^10^ vg/mouse) by subretinal injection, and the severity score was monitored during disease development (**Figure 6A**). After the incidence of the highest score between these two groups was analyzed, an almost 25% reduction in the disease score in mice treated with rAAV.shTLR7 compared with mice treated with rAAV.sc was observed (**Figure 6B**). No mice with severe disease (score≥3) were observed in the rAAV.shTLR7-treated group, while 40% were present in the rAAV.sc-treated group (**Figure 6B**). Histological analysis and fundoscopy examination also revealed disease lesions with improved pathological morphology and lower cell infiltration in ocular after being treated with rAAV.shTLR7 (**Figures 6C, D**). In addition, compared with mice in the rAAV.sc group, mice treated with rAAV.shTLR7 exhibited reduced production of the pathogenic uveitis cytokines IFN-γ and IL-17 by splenocytes in response to IRBP (**Figure 6E**), although no significant difference was found in the production of IL-10 (**Figure 6F**) or Treg frequency (**Figure 6G**). Similar results were found in aEAU animal model. The aEAU mice treated with rAAV.shTLR7 showed lower *tlr7* gene expression in the RPE layers and disease severity (**Supplementary Figures 4A, B**). Histological analysis and fundoscopy examination indicated improved pathology after being treated with rAAV.shTLR7 (**Supplementary Figures 4C, D**). Reduction of pathogenic cytokines IFN-γ and IL-17 were observed in splenocytes in response to IRBP in rAAV.shTLR7 group (**Supplementary Figure 4E**), No significant difference in Treg frequency and/or IRBP-specific Treg response was found in these groups of mice (data not shown). Collectively, the above results in aEAU and tEAU models suggested that TLR7-mediated responses were involved in the underlying mechanism contributing to inflammation and disease development.

## Discussion

In this study, we revealed the significance of TLR7 signaling in the uveopathogenic inflammatory response and maintaining the barrier function of RPE cells. First, we demonstrated different patterns of *tlr* expression in aEAU and tEAU. To avoid the effects of adjuvants that serve as TLR ligands, the tEAU model was used throughout the study. Compared with that in healthy controls, *tlr4* expression was elevated only in aEAU mice but not in tEAU mice (**Figure 1C**), indicating that the induction of *tlr4* expression might result from the presence of adjuvants during the induction of aEAU. It was indeed reported that MTB, a major component of CFA, could trigger an immune response through TLR2, TLR4 and TLR9 (24, 41); moreover, PTX appeared to not only enhance the Th1 response but also stimulate TLR4 in DCs (42, 43).

Significantly increased levels of *tlr7* and decreased *tlr3* expression were found in the aEAU and tEAU groups (**Figure 1C**). It is well known that APCs stimulated with immunogenic DAMPs might cause immune-mediated inflammatory diseases (IMIDs) (44), including autoimmune diseases (45, 46). The epicutaneous application of imiquimod, a TLR7 agonist, was able to induce acute cutaneous inflammation, and plasmacytoid dendritic cells (pDCs) acted as primary sensors. The inflammatory reaction was initiated by pDCs and showed a deviation toward the IL-23/TH17 axis, the typical molecular signature of human psoriasis (47). Moreover, endogenous nucleic acids have been shown to activate DCs and autoreactive B cells *via* TLR7 signaling, resulting in the development of SLE (48, 49). For TLR7-mediated T cell responses, activation of TLR7 signaling appeared to inhibit the differentiation of Th1 and Th17 cells and thus ameliorated the development and disease severity in the experimental autoimmune encephalomyelitis (EAE) animal model (50).

In addition, single-stranded RNA (ssRNA) is the natural ligand of TLR7, and worldwide pandemic diseases such as flu and COVID-19 are all caused by RNA viruses (51, 52). S Diebold et al. showed that innate responses to ssRNA, such as that of influenza virus, were critically dependent on TLR7, and this response required endosomal recognition of influenza genomic RNA, which then induced TLR7-dependent production of inflammatory cytokines (51). In a recent clinical trial, the combination of adjuvants containing TLR7/8 agonists and inactive SARS-CoV-2 virus enhanced the IgG2a/IgG1 ratio and Th1-biased immunity mediated by antigen-specific IFN-γ-expressing helper T cells, which provided longer protective immunity (53, 54).

Regarding the role of TLR3 signaling, although Fang et al. demonstrated that subcutaneous injection of TLR2, 3, 4, and 9 agonists could exacerbate uveitis, knockout of these genes in mice except MyD88 still resulted in the development of uveitis with similar severity (23), indicating that MyD88 but not TLR2, 3, 4 or 9 is essential for the induction of EAU. In our study, although there appeared to be no elevated levels of *tlr3* in both EAU animal models *in vivo*, the basal level of *tlr3* was much higher than *tlr7* **Figure 1C**). In fact, S Chen et al. targeted *tlr3* and also demonstrated a successful therapeutic strategy in treating EAU by chitosan-loaded TLR3-siRNA, according to the result of the induced *tlr3* level in primary RPE cells by TLRs’ agonist *in vitro* (55), suggesting the involvement of TLR3 in the development of EAU.

Accordingly, TLR7 expression appears to serve as a potential target for manipulating the inflammatory responses in EAU. In this study, local delivery of the TLR7 agonist to tEAU mice significantly enhanced disease severity, which was followed by elevated *tlr*7 expression in RPE cells (**Figures 2A–C**). Additionally, in R848-treated tEAU mice, there appeared to be fewer CD4^+^CD25^+^ and CD4^+^CD25^+^Foxp3^+^ Tregs (**Figure 3E**) as well as reduced levels of the regulatory cytokine IL-10 produced by splenocytes in response to the autoantigen IRBP (**Figure 3D**), indicating the induction of regulatory responses in an antigen-specific manner. Although the expression of *tgf-β* was also increased in the R848-treated group (**Figure 3B**), this factor was considered to collaborate with IL-6, which was induced by R848 (**Figures 3A, C**), and consequently enhanced the production of IL-17 (56, 57). This result suggested that R848 could exacerbate Th17-mediated inflammation in EAU mice.

Previous studies have shown that IL-17 and other Th17 cytokines significantly impair the function of the epithelial barrier in the airway (58). In Sjogren’s syndrome, IL-17 secreted by infiltrating lymphocytes disrupts the integrity of the tight junction barrier by downregulating claudin-4 and ZO-1 expression in submandibular glands (59). Moreover, IL-17 treatment resulted in the impairment of barrier function (**Figures 4A, B**), which was consistent with a previous study that showed that IL-17 profoundly disturbed the distribution of tight function proteins (TJPs) and consequently affected the barrier function of ARPE-19 cells (40). Similarly, R848 treatment affected barrier function in primary mouse RPE cells (**Figures 4C, D**), which may be a result of reduced TJP expression through an R848-induced IL-17 response (**Figure 4E**). Moreover, there appeared to be upregulation of Akt and NF-κB (**Supplementary Figure 5**), which are known to be positively associated with the loss of barrier function in epithelial cells (60). Additionally, R848 may have more effects than the production of immune-associated molecules and the impairment of barrier function of RPE cells. Indeed, the R848-induced responses in RPE cells also include an increase in ERK/CREB and the upregulation of STAT5 (**Supplementary Figure 5**). The former is known to positively correlate with reactive oxygen species (ROS)-mediated damage to RPE cells in age-related macular degeneration (AMD) (61), and the latter has been shown to contribute to the proliferation, differentiation and apoptosis of hematopoietic cells (62). These results suggested that several transcription factors involved in signaling pathways might be important in R848-induced responses in RPE cells and contribute to inflammatory responses in autoimmune uveitis.

Chloroquine, an antagonist of TLR7, has been used clinically to treat malaria infection since World War II (63). In fact, chloroquine is also commonly used to treat several autoimmune diseases, such as SLE, RA and Sjogren’s syndrome (64–66). In our study, TLR7 antagonist HCQ appeared to reduce disease severity slightly in tEAU mice (data not shown). However, retinal toxicity and macular retinopathy are well-known side effects of the TLR7 antagonist HCQ (67), a derivative of chloroquine. This finding led us to adapt an RNAi strategy to reduce/interfere with TLR7 signaling *via* gene therapy combined with an rAAV delivery system (**Figure 5**). The results might not be very promising, but they indeed showed the possibility of ameliorating disease severity and suppressing autoreactive immune responses in both tEAU and aEAU (**Figure 6** and **Supplementary Figure 4**). It was also noted that TLR7 expression was extremely low not only in the retinal pigment epithelium but also in primary RPE cells (**Supplementary Figure 6**) when we attempted to validate the efficacy of pAAV-shTLR7 *in vitro*. In fact, to confirm the suppressive effect of pAAV-shTLR7, we further used the ID8 cell line, which was accidentally found to have increased *tlr7* expression, to assess the efficiency of *tlr7* inhibition.

**Figure 7:**
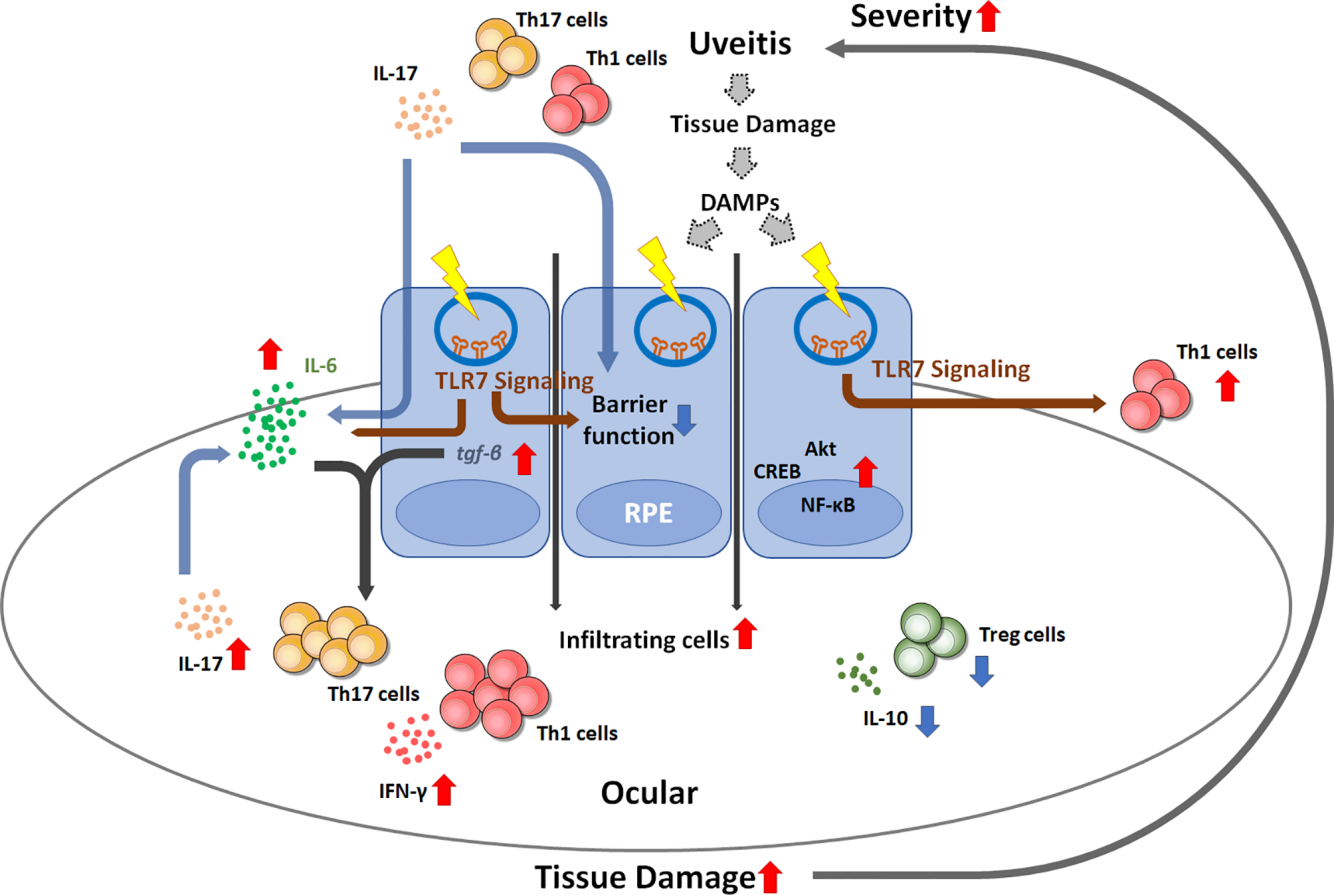
Schematic diagram showing the possible mechanism by which TLR7 signaling contributes to uveitis disease progression. Once uveitis develops and progresses, autoreactive T cells can trigger TLR7 signaling in RPE cells by direct stimulation or DAMP-induced signaling. TLR7 signaling activation results in impaired barrier function, enhanced IL-6 production and inflammatory signaling activation, which consequently enhances Th1 and Th17 differentiation and reduces regulatory T cells. The resultant increase in IL-17 may impair the barrier function of RPE cells. These events can create a positive feedback loop and lead to the exacerbation of uveitis inflammation.

In summary, our results elucidate the role of TLR7 signaling in EAU pathogenesis by affecting the characteristics of RPE and pathogenic T cells in uveitis. Pathogenic T cells might shape RPE cells and induce higher TLR7 expression through direct or indirect pathways by the stimulation of DAMPs that might have been produced by T cell-mediated tissue damage in uveitis. TLR7 signaling appears to induce increased IL-6 production by RPE cells and skew T cells toward more aggressive uveopathogenic phenotypes with more Th1 and Th17 cells and fewer Tregs. Moreover, TLR7 signaling also affects the barrier function of RPE cells, and such impaired BRB function in uveitis results in a positive feedback loop with increased pathogenic T cell infiltration in the ocular environment, leading to the exacerbation of uveitis severity (**Figure 7**). Therefore, TLR7 signaling changes RPE cells from a protective cell type toward an inflammatory enhancer and impairs barrier function. These findings provide a possible mechanism that results in autoimmune uveitis and a potential therapeutic strategy.

## Data Availability Statement

The original contributions presented in the study are included in the article/**Supplementary Material**. Further inquiries can be directed to the corresponding author.

## Ethics Statement

The animal study was reviewed and approved by Institutional Animal Care and Use Committee of Chang Gung University, Taiwan.

## Author Contributions

S-ML and C-RS conceived and designed the experiments. S-ML, Y-SH, W-HH, W-CY, and M-HL performed the experiments. S-ML and C-RS analyzed the data. Y-SH, C-LL, C-NS, and C-RS contributed to the protocol, reagents, materials and analysis tools. S-ML and C-RS wrote the manuscript. All authors contributed to the article and approved the submitted version.

## Funding

This study was financially supported by grants from Chang Gung Memorial Hospital (CMRPD1I0071-2, CMRPD1K0491-2 and BMRP440) and from the Taiwan Ministry of Science and Technology (MOST 110-2320-B-182-018).

## Conflict of Interest

The authors declare that the research was conducted in the absence of any commercial or financial relationships that could be construed as a potential conflict of interest.

## Publisher’s Note

All claims expressed in this article are solely those of the authors and do not necessarily represent those of their affiliated organizations, or those of the publisher, the editors and the reviewers. Any product that may be evaluated in this article, or claim that may be made by its manufacturer, is not guaranteed or endorsed by the publisher.
