# Supplementary material for: Inhibiting TLR7 Expression in the Retinal Pigment Epithelium Suppresses Experimental Autoimmune Uveitis
Source: Front Immunol. 2022 Jan 5;12:736261. doi: 10.3389/fimmu.2021.736261 (PMC8766412; doi:10.3389/fimmu.2021.736261)
Supplement: Supplementary file 1 [file DataSheet_1.pdf]

Supplementary Material

Inhibiting TLR7 Expression in the Retinal Pigment Epithelium Suppresses Experimental Autoimmune Uveitis

Sheng-Min Lo, Yih-Shiou Hwang, Chao-Lin Liu, Chia-Ning Shen, Wei-Hsin Hong, Wei-Cheng Yang, Meng-Hua Lee, Chia-Rui Shen

*** Correspondence:** Chia-Rui Shen: crshen@mail.cgu.edu.tw

Supplementary Figures

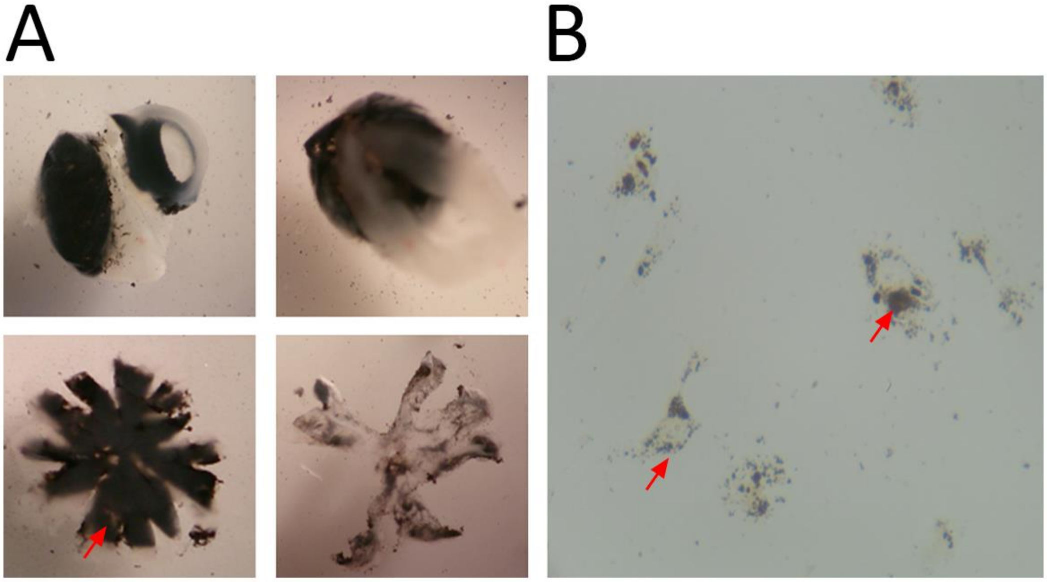

Supplementary Figure 1. Images showing the pigment in freshly isolated and cultured primary murine RPE cells.

(A) Images showing the different stages of primary RPE cell isolation are shown. First, the eyeball was enucleated from infant C57BL/6 mice (10-14 days old), and the retinal layer was removed from the eyecup. After the eyecup was treated with trypsin, the RPE layer was scraped and obtained. The red arrow indicates black pigments within the tissue, and this tissue was considered the RPE layer. **(B)** Freshly isolated RPE cells were cultured in complete growth medium, and the medium was refreshed every 3 days for cell expansion until the cells reached approximately 90% confluence. On days 4-7 after isolation, adherent cells containing pigments (red arrows) could be observed by microscopy (magnification: 400×).

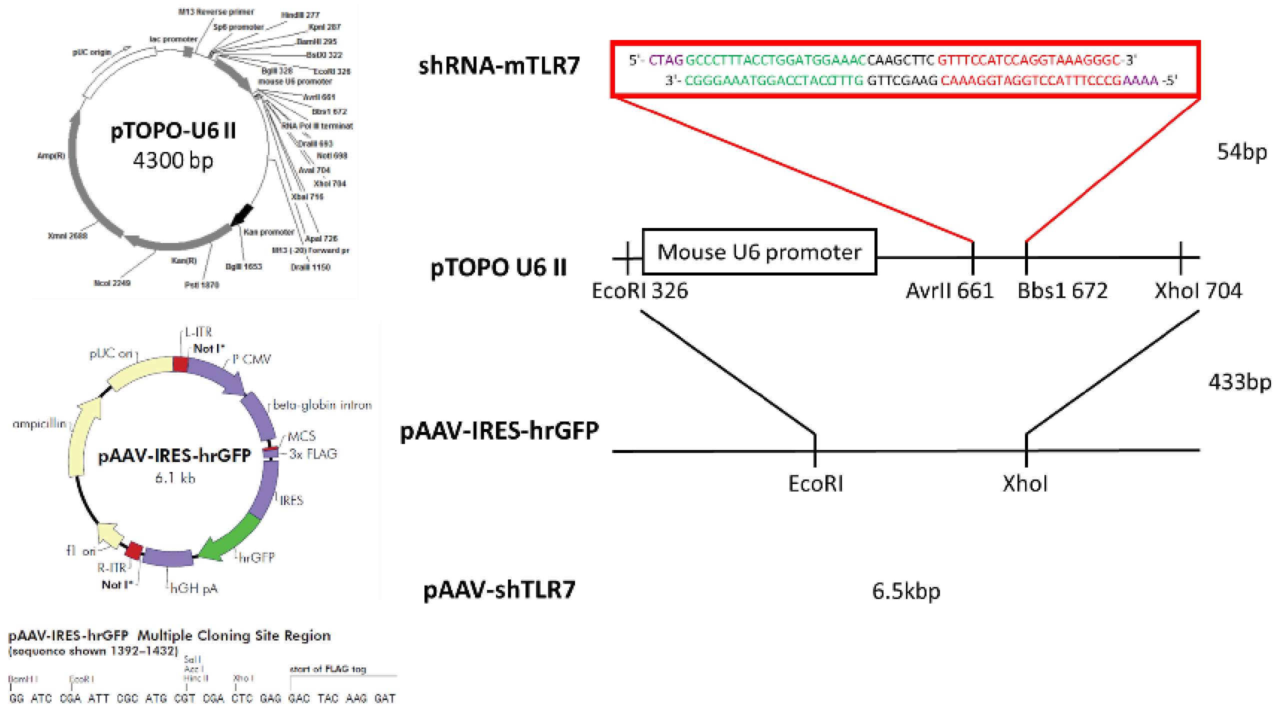

Supplementary Figure 2. Schematic construction of the pAAV.shTLR7 vector

The DNA fragment, containing the designed shRNA-TLR7 sequence within AvrII and Bbs1 cutting sites, was constructed to the pTOPO-U6 II vector. Then, the fragment of U6 promoter and shRNA-TLR7 was further constructed to pAAV-IRES-hrGFP vector via the utilization of EcoRI and XhoI.

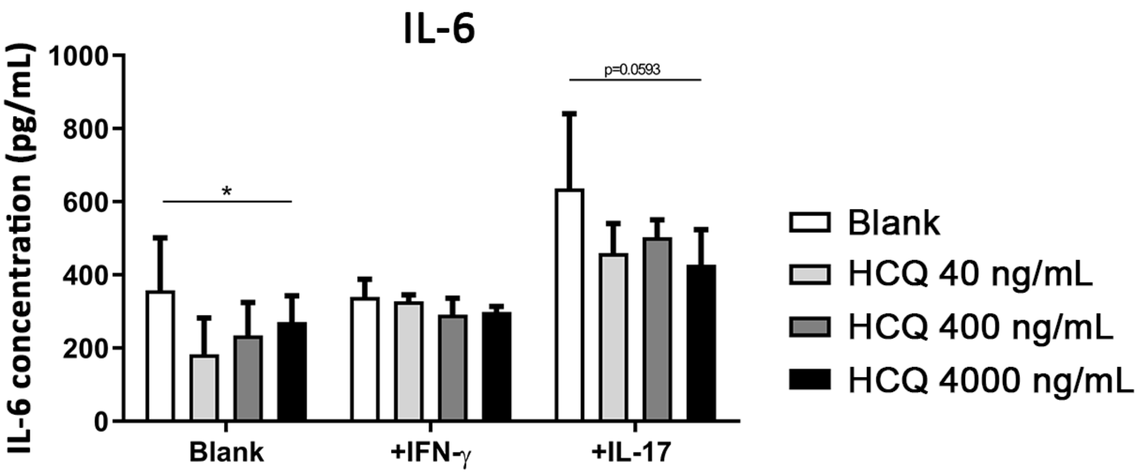

Supplementary Figure 3. Treatment with HCQ inhibited IL-6 production by primary RPE cells

Cultured primary RPE cells were stimulated with different doses of the TLR7 antagonist HCQ in the presence or absence of IFN-γ and IL-17 for 24 hours. The IL-6 levels in the culture supernatants were assayed by ELISA. The data shown are representative of at least three experiments and presented as the mean ± SD (*$p<0.05$).

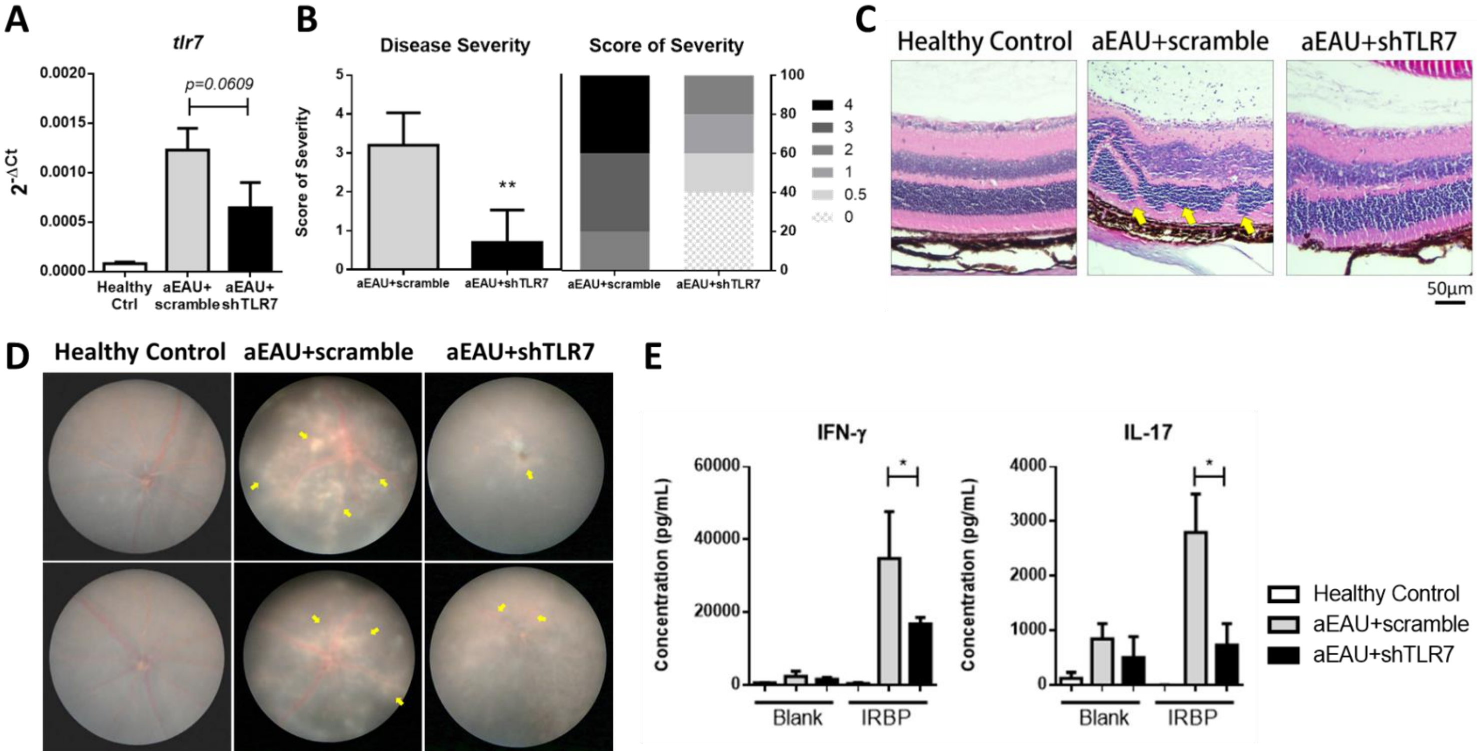

Supplementary Figure 4. Subretinal delivery of rAAV.shTLR7 reduced disease severity in the aEAU model.

C57BL/6 mice were induced to develop aEAU. On the same day, aEAU mice were subretinally injected with rAAV.shTLR7 (aEAU+shTLR7, n=5). The mice that received injections of rAAV.sc served as controls (aEAU+scramble, n=5). The mice without aEAU induction and injection of rAAV served as healthy control. On the day of sacrifice (day 16), splenocytes and one eye from each mouse were harvested for autoimmunity and histological analyses. **(A)** Primary RPE layer was freshly obtained from the animals, and tlr7 gene expression was examined by RT-qPCR. **(B)** The left panel shows the disease severity, which indicates the disease severity of individual mice, and the right panel shows the disease distribution on day 16 among the aEAU mice treated with rAAV.shTLR7 or rAAV.sc. **(C)** The eyes of aEAU mice in the two groups were fixed in 3.7% formaldehyde, sectioned and stained with H&E for ocular histopathological analysis (magnification: 200×) **(D)** The representative images showing fundoscopic analysis of aEAU mice treated with rAAV.sc or rAAV.shTLR7 on day 16 before sacrifice. **(E)** Splenocytes were obtained and cultured in the presence of IRBP (10 μg/mL), and the levels of IFN-γ and IL-17 in the culture supernatants (96 hours) were determined by ELISA. The data are presented as the mean ± SD (*p<0.05).

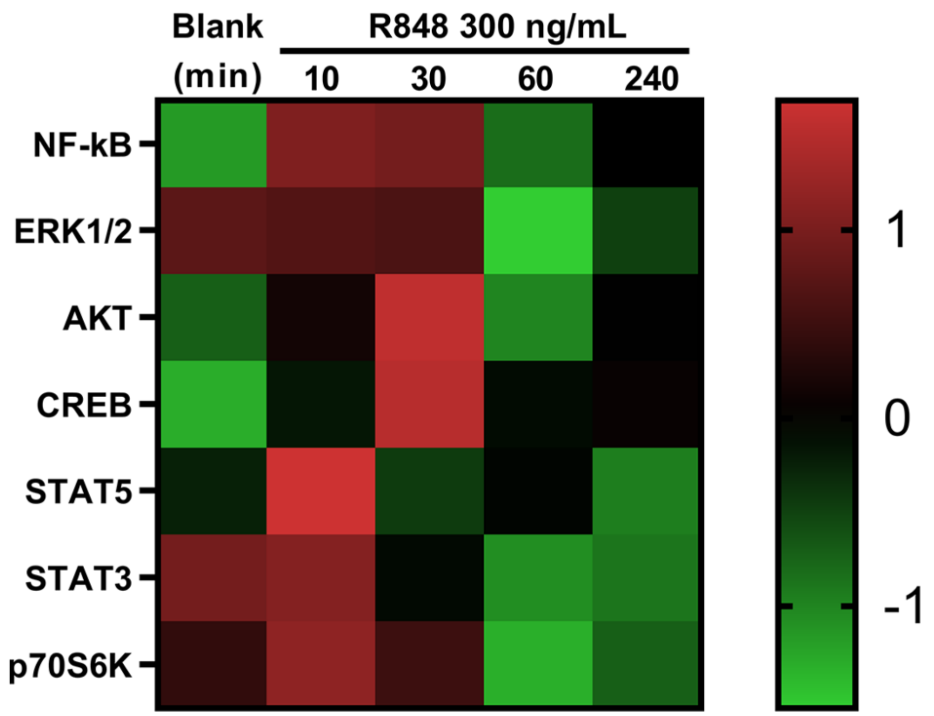

Supplementary Figure 5. The contribution of R848 to the signaling pathways in primary RPE cells.

Primary RPE cells were isolated from young C57BL/6 mice (10-14 days old), expanded and treated with R848 (300 ng/mL) for 0, 10, 30, 60 or 240 minutes. After treatment, the cellular proteins were extracted and analyzed by MILLIPLEX® Luminex assays according to the manufacturer's instructions. The data were normalized to the average of each signaling protein level and are displayed as heatmaps.

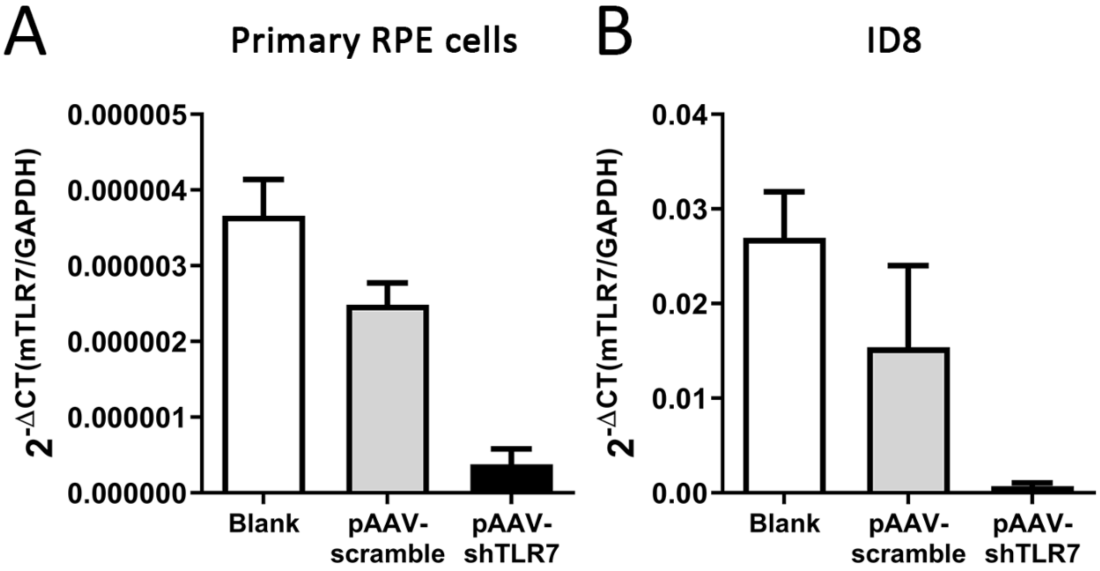

Supplementary Figure 6. TLR7 expression was suppressed in primary RPE and ID8 cells after transfection with pAAV-shTLR7.

The pAAV-shTLR7 plasmid was constructed, and its ability to suppress TLR7 expression was examined in primary RPE cells and ID8 cells, which are known to express high levels of TLR7. **(A)** Primary RPE cells and **(B)** ID8 cells were cultured and transfected with pAAV-shTLR7 and pAAV-scramble for 48 hours, RNA was extracted, and r tlr7 gene expression was analyzed by RT-qPCR. The data shown are representative of at least two experiments and presented as the mean ± SD.
